# 3-Dimensional Plasmonic Substrates Based on Chicken Eggshell Bio-Templates for SERS-Based Bio-Sensing

**DOI:** 10.3390/mi8060196

**Published:** 2017-06-21

**Authors:** Md Masud Parvez Arnob, Wei-Chuan Shih

**Affiliations:** 1 Department of Electrical and Computer Engineering, University of Houston, Houston, TX 77204, USA; arnobmasudparvez@gmail.com; 2 Department of Biomedical Engineering, University of Houston, Houston, TX 77204, USA; 3 Program of Materials Science and Engineering, University of Houston, Houston, TX 77204, USA; 4 Department of Chemistry, University of Houston, Houston, TX 77204, USA

**Keywords:** chicken eggshell, bio-inspired plasmonic substrates, low-cost SERS substrates, single bacteria detection, flexible plasmonic/SERS substrates

## Abstract

A simple technique is presented to fabricate stable and reproducible plasmonic substrates using chicken eggshell as bio-templates, an otherwise everyday waste material. The 3-dimensional (3D) submicron features on the outer shell (OS), inner shell (IS), and shell membrane (SM) regions are sputter coated with gold and characterized for surface-enhanced Raman scattering (SERS) performance with respect to coating thickness, enhancement factor (EF), hot-spots distribution, and reproducibility. The OS and IS substrates have similar EF (2.6 × 10^6^ and 1.8 × 10^6^, respectively), while the SM provides smaller EF (1.5 × 10^5^) due to its larger characteristic feature size. The variability from them (calculated as relative standard deviation, %RSD) are less than 7, 15, and 9 for the OS, IS, and SM substrates, respectively. Due to the larger EF and better signal reproducibility, the OS region is used for label-free sensing and identification of *Escherichia coli* and *Bacillus subtilis* bacteria as an example of the potential SERS applications. It is demonstrated that the detection limit could reach the level of single bacterial cells. The OS and IS regions are also used as templates to fabricate 3D flexible SERS substrates using polydimethylsiloxane and characterized. The simple, low-cost, and green route of fabricating plasmonic substrates represents an innovative alternative approach without the needs for nanofabrication facilities. Coupled with hyperspectral Raman imaging, high-throughput bio-sensing can be carried out at the single pathogen level.

## 1. Introduction

Surface-enhanced Raman scattering (SERS) has gained significant attention as an emerging analytical tool since its discovery [1,2,3,4,5]. When a molecule is placed either in the vicinity or on the surface of metallic nanostructures, the Raman scattering intensity of the molecule is enhanced by many orders of magnitude. In some cases, single-molecule sensitivity has been demonstrated [6,7]. The significantly enhanced Raman scattering of the target molecules can be mainly attributed to the local electric field (E-field) enhancement due to the excitation of localized surface plasmon resonance (LSPR) on the nanostructured metal surface [8]. Consequently, great efforts have been devoted to the fabrication of metallic nanostructures that can concentrate light into nanosized volumes with enhanced E-field [9,10,11,12].

Initial SERS works utilized chemical roughening of metal surfaces and metallic colloidal conjugates, but these were found to exhibit poor reproducibility [13,14]. Recent progress in nanoscience has led to highly reproducible techniques for fabricating complex nanostructures for SERS. These techniques include electron-beam lithography, chemical etching, colloid immobilization, annealing of metal-ion-implanted silicon, and nanosphere lithography [15,16,17]. Most of these patterning techniques still require significant laboratory infrastructures and sophisticated preparation procedures, limiting further adoption of SERS by researchers without nanofabrication expertise [18]. Hence, the identification of alternative techniques is of immense interest despite the obvious scientific challenges. Furthermore, obtaining a clean, non-toxic, and environment friendly fabrication technique is very appealing.

Recently, researchers have been inspired by naturally occurring submicron structures to develop facile, low-cost, and green SERS substrates for various applications [11,19,20,21,22,23,24,25,26]. Numerous insects, birds, and plant leaves exhibit a wide array of complex periodic and quasi-periodic micro/nanostructures, which have been exploited by coating a gold or silver thin film. The metal incorporation in naturally occurring ultrastructure can be categorized into two different directions. One promising direction is the direct replication of natural hierarchical structures by depositing a metal via physical vapor deposition (PVD). For example, Stoddart et al. used Ag (60 nm) coated cicada wing (200 nm papillary structures) as SERS substrate and obtained ~10^6^ enhancement factor [19]. Garrett et al. showed that the chitinous nanostructures (nanocone array, ~390 nm peak to peak distance) found on the wings of the Graphium butterfly can be used as SERS substrate [20]. The reported enhancement factor for Au (90 nm) and Ag (70 nm) coatings were 1.9 × 10^6^ ± 5.8 × 10^4^ and 1.6 × 10^7^ ± 1.8 × 10^4^, respectively. Xu et al. developed an efficient SERS substrate by coating silver thin film (30 nm) on natural rose petal and detected as low as 10^−9^ M drop casted Rhodamine 6G (R6G) molecules [11]. Wang et al. reported the SERS activity of carbon nanotube (CNT) (30 to 50 nm diameter) anchored chicken eggshell membrane (0.5 to 1 µm fiber width) by depositing Ag (12 min sputter deposition at 40 mA current) and detected 10^−13^ M drop casted R6G molecules [26]. Another route employed metal conjugation by decorating colloidal metallic nanoparticles on the three-dimensional (3D) framework of a natural material. For instance, Kumar et al. utilized the tip apex (300–400 nm) of Arabidopsis trichomes, a small flowering plant in the mustard family, as SERS probe by aggregating metal nanoparticles (20–100 nm) on it [22]. The calculated enhancement factor was approximately 3.22 × 10^6^. Tanahashi et al. deposited Ag nanoparticles (100–250 nm) on TiO_2_-coated cicada wings (diameters and separations of the nanopillar array are about 130 nm and 30–130 nm, respectively) to obtain a SERS substrate and reported 25 times signal enhancement when compared to thin Ag film [25]. These results suggest that plasmonic substrates, based on bio-templates, can potentially be employed in bio-sensing.

Recently, porous nanomaterials have attracted significant attention in plasmonic bio-sensing for their large surface area and high-density plasmonic hot-spots [27,28]. Eggshells are inherently porous and have been explored to produce more plasmonic hot-spots where E-field is further enhanced. Besides, the natural 3D nanostructures on eggshell provide an excellent framework for 3D SERS substrate, which has the potential to further expand the arrangement of hot-spots along the third dimension, thereby increasing the hot-spot density within the laser footprint. It is quite difficult to conveniently generate reproducible 3D structures using the traditional fabrication methods. Moreover, the eggshell is the most abundant and easily obtainable everyday waste material from kitchens and industries. Hence, eggshell is an important bio-template for SERS applications. As an example, Lin et al. deposited Ag nanoparticles (180–210 nm) on 3D porous chicken eggshell membrane and reported the SERS enhancement factor of ~10^4^ to ~10^7^ [24]. However, very few studies have been reported regarding eggshell membrane as natural SERS substrate. In addition, to our knowledge, there has been no efforts of utilizing the eggshell outer and inner surfaces, which contain 3D distributed CaCO_3_ nanospheres [29], as SERS substrates.

In this paper, we systematically investigate the SERS performance of three different eggshell regions, outer shell (OS), inner shell (IS), and shell membrane (SM), after a thin Au coating. The use of Au makes these substrates stable with highly reproducible results. As a sensor application, Gram-negative *Escherichia coli* (*E. coli*) and Gram-positive *Bacillus subtilis* (*B. subtilis*) bacteria fingerprints are detected. The strong surface enhancement effect allows the observation of Raman spectra from individual bacterial cells excited at low incident power density and short data acquisition time, thereby enabling bacterial detection without the need for time-consuming cell culture, polymerase chain reaction (PCR), or labeling [30]. In addition to being used as SERS substrates, the OS and IS regions are also used as templates for fabricating 3D flexible plasmonic/SERS substrates using polydimethylsiloxane (PDMS). The flexible SERS substrates provide the advantages of being able to be wrapped around the underlying non-planar substrates or used as swabs to collect samples from specific sites of interest. Besides, engraved plasmonic nanostructures on PDMS facilitates the integration of SERS sensor in microfluidic chip, suggesting potential point-of-care applications.

## 2. Methods

### 2.1. Preparation of Surface-Enhanced Raman Scattering (SERS) Substrates

Fresh chicken eggs, obtained from the local supermarket, were gently broken. After removing the yolk and egg white, the shells were washed with water, and the middle region was considered for the subsequent substrate preparation. The white semipermeable SM was carefully peeled and cleaned with deionized water. The clean SM was dried in air at ambient conditions and then cut into small pieces (9 to 16 mm^2^). After removing the SM, the eggshell was split into pieces of ~4 to 9 mm^2^ area, and divided into two groups. The first group was washed with deionized water, dried by nitrogen flow, and used as the OS substrates. The second group was immersed in sodium hypochlorite for 2 min to remove the residual SM, and then treated with a graded series of ethanol for dehydration. This group was used as the IS substrates. All the three different eggshell regions were sputter coated (3 × 10^−6^ Torr, Denton Desk II, Denton Vacuum LLC, Moorestown, NJ, USA) by Au of different thicknesses to obtain the SERS substrates.

### 2.2. Preparation of Bacterial Suspensions

Tryptic soy broth (TSB) was used as the growth medium for both Gram-negative *E. coli* K12 and Gram-positive *B. subtilis* strains. From a cultured bacterial stock on a trypticase soy agar (TSA) plate, an isolated colony was picked and inoculated in 20 mL TSB. This was grown overnight at 35 °C with mild shaking. The following day, bacterial cultures were centrifuged at 10,000 rpm for 5 min, and washed 3 to 4 times with sterilized deionized (DI) water. The resulting bacterial pellets were re-suspended in sterilized DI water. Optical density (OD) of the suspensions were adjusted to 0.5 at 600 nm wavelength using a Synergy MX Microtiter plate reader (Synergy 4, Biotek, Winooski, VT, USA). This OD roughly matches to a bacterial concentration of 10^7^ CFU/mL.

### 2.3. Fabrication of 3D Flexible SERS Substrates

PDMS was prepared by mixing the monomer with a base in a weight ratio of 10:1. The mixture was poured on the OS/IS region and degassed in vacuum for 10 min to promote the filling of the nanostructure. Thermal curing of the PDMS was then performed by heating on a hotplate at 60 °C for >12 h. After curing, the PDMS was peeled carefully and immersed in dilute acetic acid to remove the residual CaCO_3_ from the OS/IS template. The clean PDMS with engraved inverse image of the OS/IS 3D nanostructures was then sputter coated with 80 nm Au (3 × 10^−6^ Torr, Denton Desk II) to obtain the 3D flexible SERS substrates.

### 2.4. SERS Measurements of Benzenethiol (BT), Rhodamine 6G (R6G), and Bacteria

For Benzenethiol (BT) SERS, a self-assembled monolayer (SAM) was obtained by incubating the substrates in 4 mM BT solutions overnight. After incubation, copious amount of ethanol was used to remove the excess BT molecules from the substrate. For R6G and bacteria SERS, 3 µL solution was drop casted on the substrate and dried under ambient conditions.

### 2.5. Morphological and Molecular Imaging

Scanning electron microscopy (SEM) images were obtained using a PHILIPS FEI XL-30 FEG SEM (FEI, Hillsboro, OR, USA). ImageJ analysis was used to obtain the structural feature size. A Cary 5000 spectrophotometer was used to measure the extinction spectra. The SERS spectra were recorded by a home-built line-scan Raman microscopy system [31,32]. The excitation wavelength was 785 nm with ~23 to 28 mW power for the OS and IS samples and ~10 mW for the SM samples. The integration times were 10 s (BT SAM, R6G measurements) and 10 × 3 s (bacteria). An automated image curvature correction algorithm was employed with 5th-order polynomial background removal (when necessary) [33].

## 3. Results and Discussion

### 3.1. Morphological Characterization

**Figure 1:**
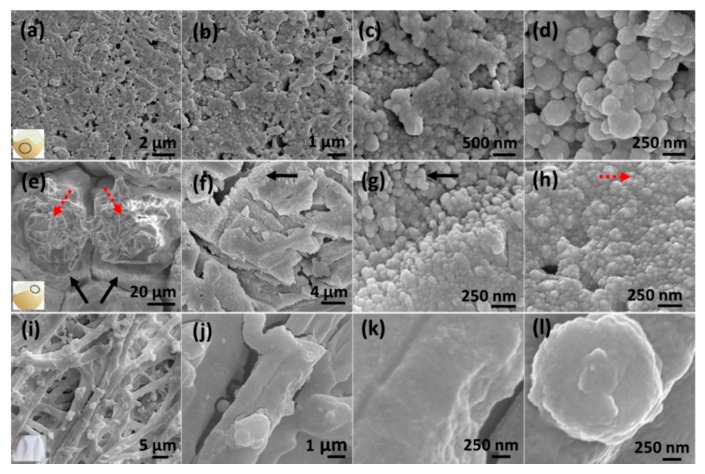
Scanning electron microscopy (SEM) images of egg (**a**–**d**) outer shell (OS) region, (**e**–**h**) inner shell (IS) region, and (**i**–**l**) shell membrane (SM) region. The OS and IS regions are coated with 80 nm Au, while the SM region is coated with 100 nm Au.

Figure 1 shows the SEM images of the three different eggshell regions, OS, IS, and SM. All these regions are sputter coated with 80 nm (OS and IS) and 100 nm (SM) Au. Figure 1a–d depict the OS region under different magnifications. The OS region consists of aggregated nanospheres of diameter ~150 to 340 nm, consistent with that reported by Zhou et al., who examined the morphology of chicken eggshell outer surface [29]. The nanospheres are randomly distributed three dimensionally with many in-between nano gaps of dimensions ranging between 50 to 100 nm (Figure 1c,d). Besides the nano gaps, there are some empty pores, ~200 to 500 nm in size, as shown in Figure 1a,b. The OS region also contains some micro cracks (~1 to 2 µm width) with missing nanospheres. The micro cracks are not shown in the SEM images since only the areas with continuous nanospheres are considered for the SERS measurements. The IS region consists of isolated island-like structures (55 to 70 µm in dimensions) as marked by the solid arrows in Figure 1e. Each island contains a 3D meshed architecture (dotted arrows) which appears as ~100 nm nanosphere aggregation as depicted in Figure 1f–h. Figure 1i–l are the SEM images of the SM region which consists of interwoven and coalescing nanofibers with multiple knobs on each fiber. The fiber diameter varies between 1.2 to 2.5 µm, where the knob diameter ranges from 0.8 to 1.5 µm. The measured SM structural dimensions are consistent with the previous reports [21,29].

### 3.2. SERS Characterization

**Figure 2:**
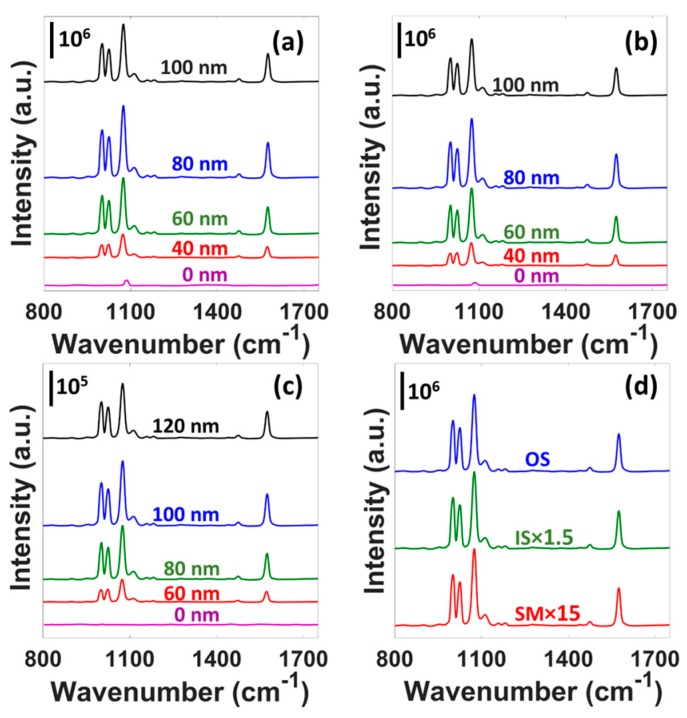
Normalized SERS spectra of 4 mM Benzenethiol (BT) self-assembled monolayer (SAM) on egg (**a**) OS, (**b**) IS, and (**c**) SM regions for different Au thicknesses. (**d**) Comparison of SERS performance between OS, IS, and SM regions.

The submicron features together with their 3D distributions allow the existence of surface plasmon resonance and plasmonic hot-spots, which exhibit strong SERS activities from these eggshell regions. Figure 2a–c display the SERS spectra of 4 mM BT SAM on the OS, IS, and SM substrates, respectively, with respect to different Au thicknesses. The SERS intensity is normalized to the incident power and integration time. Each spectrum is the average of 5 spectra from 5 different positions. For both the OS and IS substrates, SERS intensity increases up to 80 nm of deposited Au. Beyond 80 nm, the intensity starts to decline. However, the SM substrate provides the optimum SERS intensity when the deposited Au thickness is 100 nm. For both the OS and IS substrates, 80 nm Au deposition provides ~3.2 times stronger peak intensity (1076 cm^−1^) when compared to that for 40 nm Au. For the SM, the peak intensity for 100 nm Au is ~2.9 times stronger than that for the 60 nm Au. Variations in SERS intensity with respect to different metal thicknesses have also been reported in several previous studies regarding bio-inspired SERS substrates [34,35]. Thicker metal films effectively smoothen the surface nanostructures, which might cause the thickness dependent SERS performance variations. The OS, IS, and SM do not provide any BT signature peaks when there is no Au deposition (0 nm). This ensures the existence of plasmonic nanostructures and SERS phenomenon on these regions. The small peak at 1088 cm^−1^ (Figure 2a,b, 0 nm) corresponds to CaCO_3_, which is the starting material for OS and IS regions. Figure 2d shows the comparison of SERS performance between the OS (80 nm Au), IS (80 nm Au), and SM (100 nm Au) substrates. The OS substrate provides ~1.5 and 15 times stronger peak (1076 cm^−1^) intensity than that for the IS and SM substrates, respectively. The nano gaps (~50 to 100 nm) in the OS region act as strong hot-spots and provide the superior SERS performance. Although the IS region is also made of aggregated nanospheres (~100 nm diameter), their roughness and three-dimensionality are smaller compared to those for the OS region (Figure 1d,g,h). Besides, the IS region has higher non-uniformity than the OS region (Figure 1a,e). All these are plausible explanation to the slightly better SERS performance of the OS substrate than the IS one. As for the SM, the larger characteristic feature size causes its inferior SERS performance. The SERS enhancement factor (EF) for the OS, IS, and SM substrates are found to be 2.6 × 10^6^, 1.8 × 10^6^, and 1.5 × 10^5^, respectively. Details of the EF calculation is provided in the supplementary information. The calculated EFs are comparable to that for Au coated cicada (~10^6^) [19] and Graphium butterfly (1.9 × 10^6^ ± 5.8 × 10^4^) [20] wings, Au nanoparticles coated Arabidopsis plant tip (3.22 × 10^6^) [22], and Ag nanoparticles coated egg SM (~10^4^ to ~10^7^) [24].

**Figure 3:**
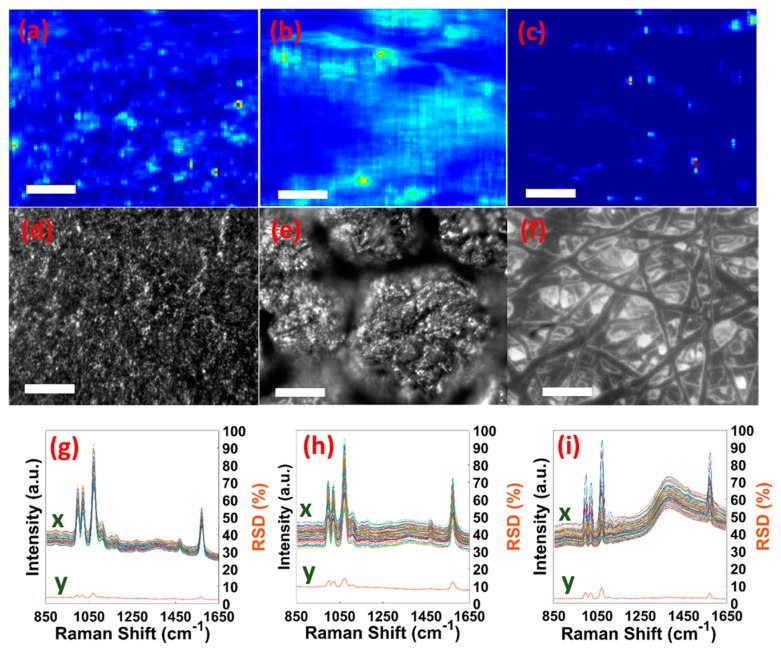
SERS maps at 1076 cm^−1^ BT peak for (**a**) OS, (**b**) IS, and (**c**) SM substrates. Typical microscopic images for (**d**) OS, (**e**) IS, and (**f**) SM substrates. The scale bar is 50 µm. %RSD-SERS graph for (**g**) OS, (**h**) IS, and (**i**) SM substrates. x denotes the 50 different BT SERS spectra and y is the corresponding %RSD values curve.

Figure 3a–c, present the SERS maps (using 1076 cm^−1^ BT peak) of 80 µm × 63 µm areas of the OS, IS, and SM substrates, respectively. The SERS map illustrates the distribution of hot-spots that contribute to the SERS signal. Figure 3d–f show the typical microscopic images (80 µm × 63 µm) of the OS, IS, and SM substrates, respectively. Although the SERS maps and the microscopic images were not taken from the same areas, they provide excellent agreement in specific morphology on various eggshell regions. The OS region is made of aggregated nanospheres, and from the microscopic image (Figure 3d), the distribution seems to be quite uniform. The hot-spots in the OS SERS map (Figure 3a) also appears to be uniformly distributed. The IS region is comprised of isolated islands with meshed network atop. The meshed network is not clear in the microscopic image (Figure 3e) due to the insufficient resolution. However, the isolated nanostructures in the IS region correlate quite well with the non-uniform hot-spots distribution in the SERS map (Figure 3b). For the SM, the hot-spots in the SERS map (Figure 3c) follow the interlacing fiber-like SM structural features, as shown in Figure 3f. The significant mapping between the SERS maps and microscopic images implies the potential of SERS as an alternative imaging technique for natural and/or fabricated nanostructures.

Figure 3g–i present the relative standard deviation (%RSD) of BT peaks to estimate the reproducibility of SERS signals on the OS, IS, and SM substrates, respectively. The %RSD values are calculated for 50 different spectra (obtained from different positions on two different samples) according to Ref. 36 [36]. Table 1 enlists the %RSD values of major BT peaks, 1003, 1025, 1076, and 1575 cm^−1^. The %RSD values are observed to be below 7, 15, and 9 for the OS, IS, and SM regions, respectively, which are comparable to that for Au nanoparticles coated Arabidopsis plant tip (5%) [22], 3D organic butterfly (Euploea mulciber) wing scales (5.2%) [37], and CNTs-anchored eggshell membrane (20%) [26].

### 3.3. Bio-Sensing Application

**Figure 4:**
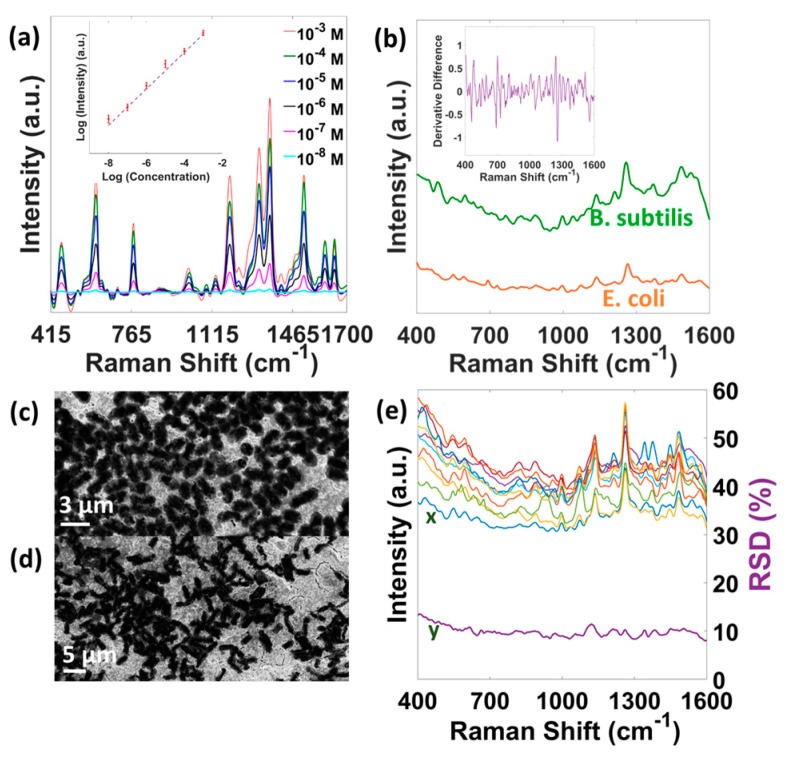
Application of egg OS SERS substrate. (**a**) SERS spectra of drop casted R6G molecules of different concentrations. Inset shows the linear variations of 1366 cm^−1^ peak intensity. (**b**) SERS spectra of *E. coli* and *B. subtilis* bacteria. Inset shows the first derivative difference of the *E. coli* and *B. subtilis* SERS spectra. SEM images of drop casted (**c**) *E. coli*, (**d**) *B. subtilis* bacteria on the OS substrate. (**e**) %RSD-SERS graph for *B. subtilis* bacterium. x denotes the 10 different SERS spectra and y is the corresponding %RSD values curve.

The larger SERS EF and better signal reproducibility make the OS region an excellent candidate for SERS based bio-molecular sensing. Figure 4a presents the SERS spectra of R6G molecules of different concentrations on the OS region. Inset shows the R6G peak intensity (at 1366 cm^−1^) variations which is almost linear in nature. The OS SERS can detect as low as 10^−8^ M concentration, or below parts per billion (ppb) level, which can potentially be useful in detecting organic pollutants in water [38]. Although bio-templated SERS substrates have been demonstrated previously, their applications in microbiology and pathogen detection has not been attempted. Figure 4b shows the unprocessed SERS spectra of Gram-negative *E. coli* and Gram-positive *B. subtilis* bacteria on the OS region. A home-built hyperspectral line-scan Raman microscope was employed where the laser was shaped into a 200 µm × 1.5 µm line with total power of ~28 mW on the sample [31]. Figure 4c,d present the SEM images of drop casted *E. coli* (3 µm × 1 µm) and *B. subtilis* (4.5 µm × 1 µm) bacteria on the OS substrate, respectively. It is observed that the bacteria SERS spectra with excellent signal-to-noise ratio (SNR) can be readily obtained when cells are placed on the Au coated OS substrate. Table 2 lists the bacterial peak assignments which are in agreement with previous reports [39,40,41,42,43]. Despite the fact that the *E. coli* and *B. subtilis* have different surface constituents, obvious spectral differences from the SERS spectra are not immediately striking. Jarvis et al. also reported similar phenomenon for *E. coli* and *B. subtilis* bacteria on borohydride reduced silver colloids [44]. However, species differentiation can be achieved using the first derivative of the SERS spectra [45]. Figure 4b inset shows the first derivative difference of the *E. coli* and *B. subtilis* SERS spectra. Some distinctive spike features are observed in the difference spectrum which implies the difference in the SERS spectra. More sophisticated statistical techniques, e.g., hierarchical cluster analysis (HCA) and/or discriminant analysis (DA) can be employed to obtain quantitative bacterial identification/classification [46,47,48]. Reproducibility has been an issue for the widespread adoption of SERS-based bacteria identification since the enhancement phenomenon depends on the microscopic morphology and stability of the SERS substrate. To address this issue, Figure 4e illustrates the spectral reproducibility of *B. subtilis* on the OS SERS substrate. The spectra are obtained from 5 different positions on the same sample and from two different samples. The corresponding relative standard deviation spectra for these data sets are also displayed in the same figure. The scattering intensities of the spectra vary by less than 12% of the mean value (at ~1135 cm^−1^), which is comparable to that for clustered gold nanoparticles covered SiO_2_ substrate (15%) [45] and Ag SERS-active substrate with high-density sub-10-nm nanogaps (14%) [49]. Similar %RSD value (less than 14) was also obtained for the *E. coli* bacteria. The %RSD value for the *B. subtilis*/*E. coli* is larger than that for the BT (6%) on the OS region. The observed difference can be attributed to the inhomogeneous bacteria aggregation on the substrate and the natural life cycle difference of the bacteria [45].

**Figure 5:**
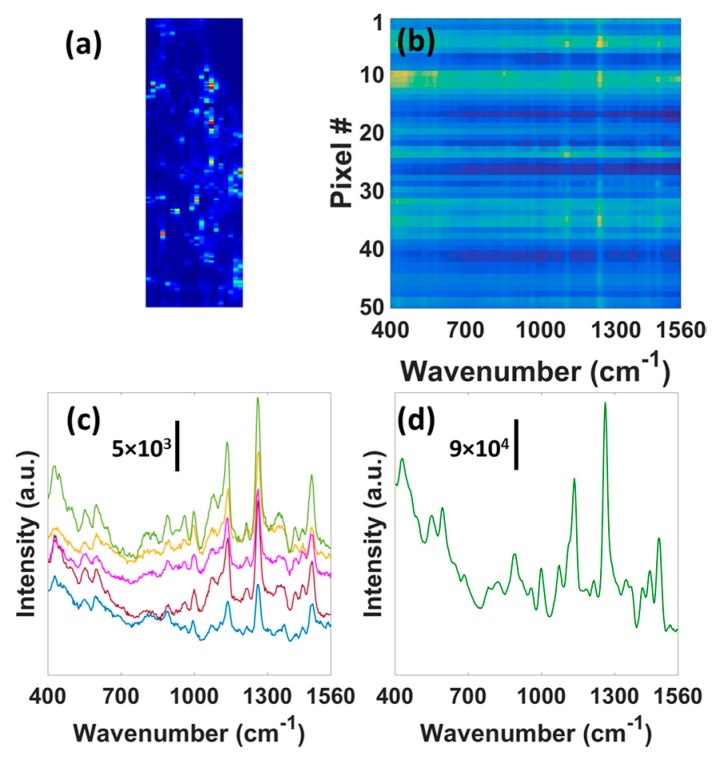
(**a**) SERS map (96 µm × 30 µm) using 1259 cm^−1^ *B. subtilis* peak. (**b**) SERS image of an 75 µm × 1.5 µm area for *B. subtilis* bacteria. Each pixel (in y direction) represents 1.5 µm length. (**c**) 5 SERS spectra corresponding to 5 different pixels (5 rows) in (b). Each pixel/row contains the SERS spectrum from the 1.5 µm × 1.5 µm area that can accommodate only half of a bacterial cell. (**d**) SERS spectrum obtained via summation of 50 pixels (contribution from ~25 bacterial cells) in (b).

Figure 5a presents the *B. subtilis* (1259 cm^−1^) SERS map of an 96 µm × 30 µm area on the OS region. The hot-spots in the map correlate quite well to the hot-spots for the BT SAM (Figure 3a). It implies that the bacteria SERS signal originates only from the surface of the OS region. Premasiri et al. also reported that only the bacterial cells close to the substrate contribute to the SERS spectra [45]. Thus, assuming the complete coverage of the bacteria on the measurement spot, a simple calculation suggests that the spectra in Figure 4b result from the SERS activity of maximum 32 (*B. subtilis*) and 48 (*E. coli*) bacterial cells. However, single bacterium detection is possible by the OS SERS substrate. Figure 5b shows the *B. subtilis* SERS image from an 75 µm × 1.5 µm area on the OS region. Each row in the image contains the SERS spectrum from the 1.5 µm × 1.5 µm area that can accommodate only half of a bacterial cell. Figure 5c presents the 5 different spectra that correspond to 5 different rows in the SERS image. All the spectra correlate quite well with the SERS spectrum in Figure 5d, obtained by summing all the 50 rows (contribution from ~25 bacterial cells) of the SERS image, indicating that the limit of detection could reach the single cell level. The ability to detect single bacterial cell implies the potential of the OS SERS substrate to identify members of a mixture of cell types, thus enabling rapid detection and identification of mixed infection components along with the study of the microbial heterogeneities.

There have been several efforts to detect single bacterial cell based on Raman spectroscopy. Schuster et al. reported the non-SERS Raman spectra of a single bacterium (*Clostridium beijerinckii*) using confocal microscopy [50]. However, they used longer illumination time (3 min) and higher incident laser power density (2.546 mW/µm^2^), as compared to the considerably low power density (93 µW/µm^2^) and short illumination time (30 s) in our study. KahRaman et al. detected single *Staphylococcus cohnii* bacterial cell based on SERS using layer-by-layer Ag nanoparticle coating of the bacterium [51]. Although the illumination time was short (10 s), the incident laser power density was still very high (3.82 mW/µm^2^). Premasiri et al. used comparable (to this study) illumination time (20 s) and incident laser power density (133 µW/µm^2^) to detect single *Bacillus anthracis* on clustered gold nanoparticles covered SiO_2_ as the SERS substrate [45]. Chu et al. reported SERS based (Ag nanorod array as the SERS substrate) single bacterium (*E. coli*) detection using very short illumination time (10 s) as well as low incident laser power density (15 µW/µm^2^) [52]. Similarly, Yang et al. used very short illumination time (10 s) and low power density (17 µW/µm^2^) to detect single *Bacillus cereus* cell using engineered Au nanoparticle cluster array as the SERS substrate [53]. However, all the previous SERS based single bacterium detection studies required sophisticated fabrication process steps to obtain the SERS substrate, whereas the OS SERS substrate is very simple to fabricate. In addition, our hyperspectral line-scan Raman microscope provides much higher throughput to acquire SERS maps from a cell population, an essential feature for real applications.

### 3.4. Eggshell for 3D Flexible SERS Substrate

**Figure 6:**
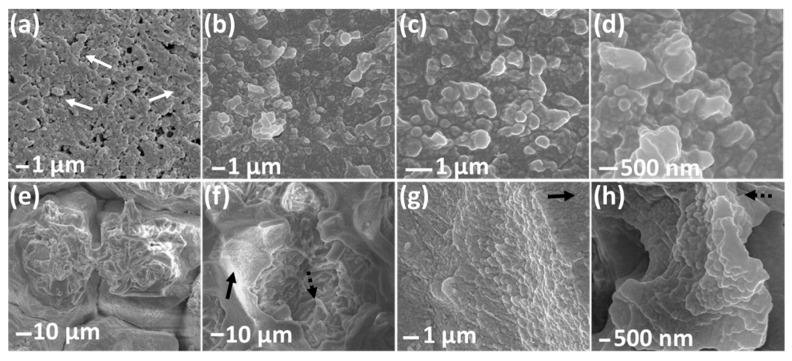
SEM image of (**a**) egg OS region, (**b**–**d**) F-i-OS substrate under different magnifications. SEM image of (**e**) egg IS region and (**f**) F-i-IS substrate. Solid and dotted arrow marks, in Figure (**f**), represent two different regions. Zoomed in SEM image of the region marked by the (**g**) solid and (**h**) dotted arrow.

Eggshells can also be used as templates for fabricating 3D flexible SERS substrates with complementary surface profile to that of the eggshells. Figure 6a,e present the SEM images of egg OS and IS regions, respectively. Figure 6b,d,f,h show the SEM images of the templated OS and IS structural features on PDMS, respectively. From the SEM images the 3D nature of the engraved PDMS substrates are quite obvious. PDMS substrates, obtained by the egg OS and IS regions, are termed as the flexible-inverse-OS (F-i-OS) and flexible-inverse-IS (F-i-IS) substrates, respectively. The F-i-OS substrate features the sub-micron structures on the OS region. The F-i-OS substrate features can be divided into two size ranges: 150 to 450 nm and 800 to 1200 nm. The origin of these different sized structural features can be understood by the architecture of the egg OS which consists of randomly distributed nanospheres (~150 to 340 nm) with many empty pores of sizes ranging between ~200 to 500 nm. The nanospheres are not distributed uniformly, rather there are some empty spaces, as marked by arrows in Figure 6a, of ~750 to 1400 nm dimensions. The nanospheres and pores give rise to 150 to 400 nm structural features, while the empty spaces yield to the larger (800 to 1200 nm) ones. The F-i-IS substrate contains the complementary surface profile of the egg IS region. Since the IS region consists of isolated island-like structures with fibrous network atop, the F-i-IS substrate contains many pit structures with fibrous network at the bottom. The fibrous network contains features with sizes ranging from 800 to 1200 nm. However, the entire structure exhibits a roughness of about ~250 to 300 nm, as shown in Figure 6g,h, which corresponds to the 3D nanoscale valleys between the aggregated nanospheres.

**Figure 7:**
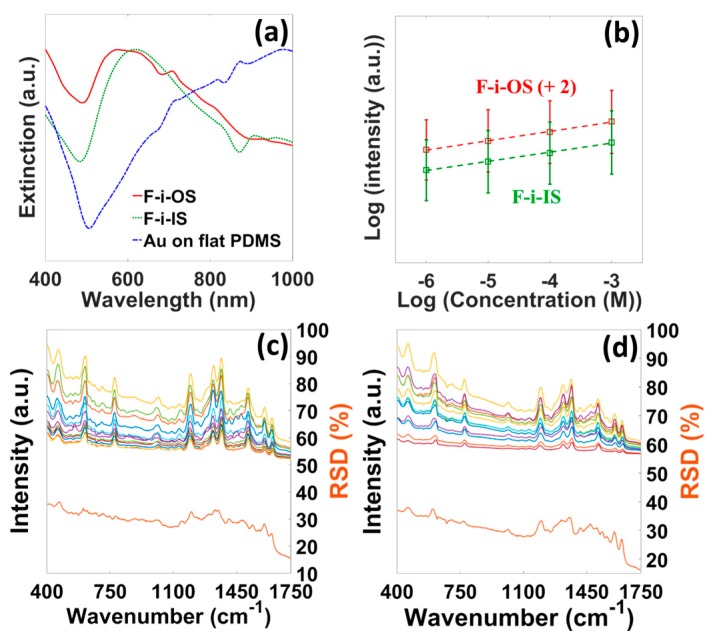
(**a**) Extinction spectra of F-i-OS/IS substrates and Au on flat PDMS. (**b**) Performance characterization of F-i-OS/IS substrates by detecting different R6G concentrations. SERS peak at 1366 cm^−1^ was considered. %RSD-SERS graph for (**c**) F-i-OS and (**d**) F-i-IS substrates. x denotes the 10 different spectra from two different samples and y is the corresponding %RSD values curve.

Figure 7a presents the extinction spectra for both F-i-OS and F-i-IS substrates and the spectrum for Au (80 nm) coated flat PDMS film. The absence of any resonance peak in the flat Au/PDMS spectrum suggests the ineffectiveness of free-space excitation of surface plasmon resonance. In contrast, broad LSPR features are identified at 580 nm and 620 nm for F-i-OS and F-i-IS substrates, respectively, providing support to the SERS activities on these substrates. Figure 7b shows the 1366 cm^−1^ peak intensity variations for different concentrations of R6G on the F-i-OS/IS substrates. Both the substrates can detect down to 10^−6^ M concentration. However, the F-i-OS substrate provides better SERS performance in terms of signal intensity (~1.8 times larger) than the F-i-IS substrate. The highly non-uniform morphology of the F-i-IS substrate might be responsible for its slightly inferior performance. Figure 7c,d present the SERS spectral reproducibility for the F-i-OS and F-i-IS substrates, respectively, calculated using 10 different spectra from two different samples. The %RSD values are below 33 and 35 for the F-i-OS and F-i-IS substrates, respectively. Shiohara et al. reported the PDMS/gold nanostar flexible substrate with comparable R6G detection limit (10^−5^ M) and better reproducibility (%RSD = 10) [54]. Wu et al. reported Ag nanoparticles (screen printed)/Polyethylene terephthalate (PET) flexible SERS substrate with better detection limit (10^−10^ M) and comparable reproducibility (%RSD = 20) [55]. Similarly, several flexible substrates have been reported in literature with either comparable or better SERS performance [56,57,58,59]. However, most of them succumb to the fabrication complexity and/or stability (due to Ag) issues. In contrast, the F-i-OS/IS substrate is extremely easy to fabricate and has better stability.

## 4. Conclusions

A simple, low-cost, and green route of fabricating 3D plasmonic substrates for SERS applications is reported based on a day-to-day waste material, chicken eggshell. Three different eggshell regions, OS, IS, and SM, are coated with Au to obtain the SERS substrates, which are characterized in terms of optimum Au thickness, EF, hot-spots distribution, and reproducibility. The OS, IS, and SM provide optimum SERS performance for 80, 80, and 100 nm of deposited Au, respectively. The OS and IS SERS substrates have almost the similar EF (2.6 × 10^6^ and 1.8 × 10^6^, respectively), while the SM provides smaller SERS enhancement (1.5 × 10^5^) due to its larger characteristic feature size. The reproducibility of the OS, IS, and SM SERS substrates are quantified by calculating the %RSD and found to be less than 7, 15, and 9, respectively. As an example of the bio-sensing capability, *E. coli* and *B. subtilis* bacteria are detected on the OS SERS substrate with excellent signal-to-noise ratio. The OS SERS substrate is capable of detecting single bacterial cell with very low incident power density and short illumination time. The OS and IS regions are also used to obtain 3D flexible SERS substrates, which are termed as F-i-OS and F-i-IS substrates, respectively. Both the F-i-OS/IS substrates can detect up to 10^−6^ M R6G with %RSD values less than 33 and 35, respectively. We believe that the reported simple, cost-effective, and green technique to obtain easy-to-access 3D SERS substrates could pave the way for widespread application of SERS-based bioassays. Coupled with hyperspectral Raman imaging, high-throughput bio-sensing can be carried out at the single pathogen level.

## Figures and Tables

**Table 1:** %RSD values of 1003, 1025, 1076, and 1575 cm^−1^ BT peaks for OS, IS, and SM substrates.

Peak Position (cm^−1^)	1003	1025	1076	1575
%RSD (OS)	4.98	5.02	6.07	4.09
%RSD (IS)	13.23	12.74	14.5	12.32
%RSD (SM)	5.87	5.64	8.83	5.67

**Table 2:** Wavenumber assignments for the bacterial SERS spectra.

Raman Shift (cm^−1^)	Assignment
550	Proteins, S–S stretch
600	Monosubstituted benzenes, CCC ring deformation
632	Proteins (tyrosine), C–S stretching and C–C twisting
666	Guanine, (C–S)
707	Adenine (nucleic acids)
732	Adenine, trans conformation of (C–S) or tryptophan
794	Cytosine, uracil (ring, stretch) (nucleic acids)
830	O–P–O stretching or tyrosine
890	Carbohydrates, COC stretch
930	Carbohydrates, C–COO stretch
963	C=C stretch or tyrosine
1000	Phenylalanine (the symmetric ring breathing mode) (proteins)
1037	Phenylalanine (the in-plane C–H bending mode) (proteins)
1079	Phenylalanine
1107	CC skeletal and COC stretch, glycosidic link (carbohydrates)
1138	Carbohydrates, C–N and C–C stretch
1163	2-Methyltetradecanoic acid or 15-Methylpalmitic acid
1264	Cytosine, adenine(DNA), amide III random coil
1343	(C–H_2_) deformation, or tryptophan
1369	Not specified
1449	Lipids, C–H deformation
1485	(C–H_2_) (protein)
1529	Adenine, cytosine, guanine
1560	Amide II, NH deformation and CN stretching

## Supplementary Materials

The following are available online at http://www.mdpi.com/2072-666X/8/6/196/s1, Figure S1: Representative OS and SM architectures; calculated E-field distribution for OS and SM architectures; the incident wavelength is 785 nm, Table S1: Calculation of SERS EF for egg OS, IS, and SM regions.

## References

[1] Albrecht M.G., Creighton J.A. (1977). Anomalously intense Raman spectra of pyridine at a silver electrode. J. Am. Chem. Soc..

[2] Kneipp K., Kneipp H., Itzkan I., Dasari R.R., Feld M.S. (1999). Ultrasensitive chemical analysis by Raman spectroscopy. Chem. Rev..

[3] Lyandres O., Shah N.C., Yonzon C.R., Walsh J.T., Glucksberg M.R., Van Duyne R.P. (2005). Real-time glucose sensing by surface-enhanced Raman spectroscopy in bovine plasma facilitated by a mixed decanethiol/mercaptohexanol partition layer. Anal. Chem..

[4] Levin C.S., Bishnoi S.W., Grady N.K., Halas N.J. (2006). Determining the conformation of thiolated poly (ethylene glycol) on au nanoshells by surface-enhanced Raman scattering spectroscopic assay. Anal. Chem..

[5] Yang S., Dai X., Stogin B.B., Wong T.-S. (2016). Ultrasensitive surface-enhanced Raman scattering detection in common fluids. Proc. Natl. Acad. Sci. USA.

[6] Nie S., Emory S.R. (1997). Probing single molecules and single nanoparticles by surface-enhanced Raman scattering. Science.

[7] Qi J., Zeng J., Zhao F., Lin S.H., Raja B., Strych U., Willson R.C., Shih W.-C. (2014). Label-free, in situ SERS monitoring of individual DNA hybridization in microfluidics. Nanoscale.

[8] Lee S.J., Morrill A.R., Moskovits M. (2006). Hot spots in silver nanowire bundles for surface-enhanced Raman spectroscopy. J. Am. Chem. Soc..

[9] Chen Y.-H., Huang L., Gan L., Li Z.-Y. (2012). Wavefront shaping of infrared light through a subwavelength hole. Light Sci. Appl..

[10] Wang L., Xiong W., Nishijima Y., Yokota Y., Ueno K., Misawa H., Qiu J., Bi G. (2011). Spectral properties of nanoengineered Ag/Au bilayer rods fabricated by electron beam lithography. Appl. Opt..

[11] Xu B.B., Zhang Y.L., Zhang W.Y., Liu X.Q., Wang J.N., Zhang X.L., Zhang D.D., Jiang H.B., Zhang R., Sun H.B. (2013). Silver-coated rose petal: Green, facile, low-cost and sustainable fabrication of a SERS substrate with unique superhydrophobicity and high efficiency. Adv. Opt. Mater..

[12] Shih W.-C., Santos G.M., Zhao F., Zenasni O., Arnob M.M.P. (2016). Simultaneous chemical and refractive index sensing in the 1–2.5 μm near-infrared wavelength range on nanoporous gold disks. Nano Lett..

[13] Talley C.E., Jackson J.B., Oubre C., Grady N.K., Hollars C.W., Lane S.M., Huser T.R., Nordlander P., Halas N.J. (2005). Surface-enhanced Raman scattering from individual Au nanoparticles and nanoparticle dimer substrates. Nano Lett..

[14] Schwartzberg A.M., Grant C.D., Wolcott A., Talley C.E., Huser T.R., Bogomolni R., Zhang J.Z. (2004). Unique gold nanoparticle aggregates as a highly active surface-enhanced Raman scattering substrate. J. Phys. Chem. B.

[15] Felidj N., Aubard J., Levi G., Krenn J., Hohenau A., Schider G., Leitner A., Aussenegg F. (2003). Optimized surface-enhanced Raman scattering on gold nanoparticle arrays. Appl. Phys. Lett..

[16] Haynes C.L., Van Duyne R.P. (2001). Nanosphere lithography: A versatile nanofabrication tool for studies of size-dependent nanoparticle optics. J. Phys. Chem. B.

[17] Fan M., Andrade G.F., Brolo A.G. (2011). A review on the fabrication of substrates for surface enhanced Raman spectroscopy and their applications in analytical chemistry. Anal. Chim. Acta.

[18] Stiles P.L., Dieringer J.A., Shah N.C., Van Duyne R.P. (2008). Surface-enhanced Raman spectroscopy. Ann. Rev. Anal. Chem..

[19] Stoddart P., Cadusch P., Boyce T., Erasmus R., Comins J. (2006). Optical properties of chitin: Surface-enhanced Raman scattering substrates based on antireflection structures on cicada wings. Nanotechnology.

[20] Garrett N.L., Vukusic P., Ogrin F., Sirotkin E., Winlove C.P., Moger J. (2009). Spectroscopy on the wing: Naturally inspired SERS substrates for biochemical analysis. J. Biophotonics.

[21] Zheng B., Qian L., Yuan H., Xiao D., Yang X., Paau M.C., Choi M.M. (2010). Preparation of gold nanoparticles on eggshell membrane and their biosensing application. Talanta.

[22] Kumar G.P. (2011). Gold nanoparticle-coated biomaterial as SERS micro-probes. Bull. Mater. Sci..

[23] Tan Y., Zang X., Gu J., Liu D., Zhu S., Su H., Feng C., Liu Q., Lau W.M., Moon W.-J. (2011). Morphological effects on surface-enhanced Raman scattering from silver butterfly wing scales synthesized via photoreduction. Langmuir.

[24] Lin P.Y., Hsieh C.W., Tsai P.C., Hsieh S. (2014). Porosity-controlled eggshell membrane as 3D SERS-active substrate. ChemPhysChem.

[25] Tanahashi I., Harada Y. (2015). Silver nanoparticles deposited on TiO_2_-coated cicada and butterfly wings as naturally inspired SERS substrates. J. Mater. Chem. C.

[26] Wang M., Meng G., Huang Q., Tang H., Li Z., Zhang Z. (2015). CNTs-anchored eggshell membrane decorated with Ag-NPs as cheap but effective SERS substrates. Sci. China Mater..

[27] Zeng J., Zhao F., Li M., Li C.-H., Lee T.R., Shih W.-C. (2015). Morphological control and plasmonic tuning of nanoporous gold disks by surface modifications. J. Mater. Chem. C.

[28] Jeong J.W., Arnob M.M.P., Baek K.M., Lee S.Y., Shih W.C., Jung Y.S. (2016). 3D cross-point plasmonic nanoarchitectures containing dense and regular hot spots for surface-enhanced Raman spectroscopy analysis. Adv. Mater..

[29] Zhou J., Wang S., Nie F., Feng L., Zhu G., Jiang L. (2011). Elaborate architecture of the hierarchical hen’s eggshell. Nano Res..

[30] Patel I., Premasiri W., Moir D., Ziegler L. (2008). Barcoding bacterial cells: A SERS-based methodology for pathogen identification. J. Raman Spectrosc..

[31] Qi J., Shih W.-C. (2012). Parallel Raman microspectroscopy using programmable multipoint illumination. Opt. Lett..

[32] Narendran S., Ji Q., Eric D.Y., Alexander J.L., Dina C.L., Raphael E.P., Kirill V.L., Wei-Chuan S. (2014). Line-scan Raman microscopy complements optical coherence tomography for tumor boundary detection. Laser Phys. Lett..

[33] Qi J., Bechtel K.L., Shih W.-C. (2014). Automated image curvature assessment and correction for high-throughput Raman spectroscopy and microscopy. Biomed. Spectrosc. Imaging.

[34] Tan Y., Gu J., Xu L., Zang X., Liu D., Zhang W., Liu Q., Zhu S., Su H., Feng C. (2012). High-density hotspots engineered by naturally piled-up subwavelength structures in three-dimensional copper butterfly wing scales for surface-enhanced Raman scattering detection. Adv. Funct. Mater..

[35] Jiwei Q., Yudong L., Ming Y., Qiang W., Zongqiang C., Wudeng W., Wenqiang L., Xuanyi Y., Jingjun X., Qian S. (2013). Large-area high-performance SERS substrates with deep controllable sub-10-nm gap structure fabricated by depositing Au film on the cicada wing. Nanoscale Res. Lett..

[36] Zhang B., Wang H., Lu L., Ai K., Zhang G., Cheng X. (2008). Large-area silver-coated silicon nanowire arrays for molecular sensing using surface-enhanced Raman spectroscopy. Adv. Funct. Mater..

[37] Tan Y., Gu J., Zang X., Xu W., Shi K., Xu L., Zhang D. (2011). Versatile fabrication of intact three-dimensional metallic butterfly wing scales with hierarchical sub-micrometer structures. Angew. Chem. Int. Ed..

[38] Chen J., Su H., You X., Gao J., Lau W.M., Zhang D. (2014). 3D TiO_2_ submicrostructures decorated by silver nanoparticles as SERS substrate for organic pollutants detection and degradation. Mater. Res. Bull..

[39] Su L., Zhang P., Zheng D.-W., Wang Y.-J.-Q., Zhong R.-G. (2015). Rapid detection of Escherichia coli and salmonella typhimurium by surface-enhanced Raman scattering. Optoelectron. Lett..

[40] Zhou H., Yang D., Ivleva N.P., Mircescu N.E., Niessner R., Haisch C. (2014). SERS detection of bacteria in water by in situ coating with Ag nanoparticles. Anal. Chem..

[41] Fan C., Hu Z., Mustapha A., Lin M. (2011). Rapid detection of food-and waterborne bacteria using surface-enhanced Raman spectroscopy coupled with silver nanosubstrates. Appl. Microbiol. Biotechnol..

[42] Liu Y., Chen Y.-R., Nou X., Chao K. (2007). Potential of surface-enhanced Raman spectroscopy for the rapid identification of Escherichia coli and Listeria monocytogenes cultures on silver colloidal nanoparticles. Appl. Spectrosc..

[43] Jarvis R.M., Brooker A., Goodacre R. (2004). Surface-enhanced Raman spectroscopy for bacterial discrimination utilizing a scanning electron microscope with a Raman spectroscopy interface. Anal. Chem..

[44] Jarvis R.M., Goodacre R. (2008). Characterisation and identification of bacteria using SERS. Chem. Soc. Rev..

[45] Premasiri W., Moir D., Klempner M., Krieger N., Jones G., Ziegler L. (2005). Characterization of the surface enhanced Raman scattering (SERS) of bacteria. J. Phys. Chem. B.

[46] Jarvis R.M., Goodacre R. (2004). Discrimination of bacteria using surface-enhanced Raman spectroscopy. Anal. Chem..

[47] Yang D., Zhou H., Haisch C., Niessner R., Ying Y. (2016). Reproducible *E. coli* detection based on label-free SERS and mapping. Talanta.

[48] Li J., Du Y., Qi J., Sneha R., Chang A., Mohan C., Shih W.C. (2015). Raman spectroscopy as a diagnostic tool for monitoring acute nephritis. J. Biophotonics.

[49] Chen J., Qin G., Wang J., Yu J., Shen B., Li S., Ren Y., Zuo L., Shen W., Das B. (2013). One-step fabrication of sub-10-nm plasmonic nanogaps for reliable SERS sensing of microorganisms. Biosens. Bioelectron..

[50] Schuster K.C., Reese I., Urlaub E., Gapes J.R., Lendl B. (2000). Multidimensional information on the chemical composition of single bacterial cells by confocal Raman microspectroscopy. Anal. Chem..

[51] KahRaman M., Zamaleeva A.I., Fakhrullin R.F., Culha M. (2009). Layer-by-layer coating of bacteria with noble metal nanoparticles for surface-enhanced Raman scattering. Anal. Bioanal. Chem..

[52] Chu H., Huang Y., Zhao Y. (2008). Silver nanorod arrays as a surface-enhanced Raman scattering substrate for foodborne pathogenic bacteria detection. Appl. Spectrosc..

[53] Yang L., Yan B., Premasiri W.R., Ziegler L.D., Negro L.D., Reinhard B.M. (2010). Engineering nanoparticle cluster arrays for bacterial biosensing: The role of the building block in multiscale SERS substrates. Adv. Funct. Mater..

[54] Shiohara A., Langer J., Polavarapu L., Liz-Marzán L.M. (2014). Solution processed polydimethylsiloxane/gold nanostar flexible substrates for plasmonic sensing. Nanoscale.

[55] Wu W., Liu L., Dai Z., Liu J., Yang S., Zhou L., Xiao X., Jiang C., Roy V.A. (2015). Low-cost, disposable, flexible and highly reproducible screen printed SERS substrates for the detection of various chemicals. Sci. Rep..

[56] Polavarapu L., Liz-Marzán L.M. (2013). Towards low-cost flexible substrates for nanoplasmonic sensing. Phys. Chem. Chem. Phys..

[57] Chung A.J., Huh Y.S., Erickson D. (2011). Large area flexible SERS active substrates using engineered nanostructures. Nanoscale.

[58] Singh J., Chu H., Abell J., Tripp R.A., Zhao Y. (2012). Flexible and mechanical strain resistant large area SERS active substrates. Nanoscale.

[59] Lu G., Li H., Zhang H. (2012). Gold-nanoparticle-embedded polydimethylsiloxane elastomers for highly sensitive Raman detection. Small.

