# Supplementary material for: 3-Dimensional Plasmonic Substrates Based on Chicken Eggshell Bio-Templates for SERS-Based Bio-Sensing
Source: Micromachines (Basel). 2017 Jun 21;8(6):196. doi: 10.3390/mi8060196 (PMC6190012; doi:10.3390/mi8060196)
Supplement: Supplementary file 1 [file micromachines-08-00196-s001.pdf]

Supplementary Information: 3-Dimensional Plasmonic Substrates Based on Chicken Eggshell Bio-Templates for SERS Based Bio-Sensing

Md Masud Parvez Arnob and Wei-Chuan Shih

I. Finite difference time domain (FDTD) method.

FDTD method solves Faraday's law and Ampere's law, Equations 1 and 2, in differential form over a grid-based domain (where field components are defined only at the grid points) using Taylor expansions for the derivatives.

$$\frac{\varepsilon \partial E}{\partial t} = \nabla \times H - J \qquad (1)$$

$$\frac{\varepsilon \partial H}{\partial t} = \nabla \times E \qquad (2)$$

The most popular discretization is based on Yee's algorithm and computes the E (electric field) and H (magnetic field) components at half grid points relative to each other and central spatial and leapfrog time differences are used for the derivatives [1]. A simple version of this can be generated for a 2D system in which the electric field only has x and y components, and the magnetic field has a z component. If the time step is denoted by τ, then the following equations are used:

$${E_x}^{n+1/2} = {E_x}^{n-1/2} + \frac{\tau}{\varepsilon}\left(\frac{\partial {H_z}^n}{\partial y} - {J_x}^n\right) \qquad (3)$$

$${E_y}^{n+1/2} = {E_y}^{n-1/2} + \frac{\tau}{\varepsilon}\left(\frac{\partial {H_z}^n}{\partial x} - {J_y}^n\right) \qquad (4)$$

$${H_z}^{n+1} = {H_z}^{n} + \frac{\tau}{\mu}\left(\frac{\partial {E_y}^{n+1/2}}{\partial x} - \frac{\partial {E_x}^{n+1/2}}{\partial y}\right) \qquad (5)$$

II. FDTD simulation setup.

The FDTD simulation domain was 1.6μm× 1.6 μm× 1.6 μm and 6μm× 6 μm× 6 μm for OS and SM, respectively. Perfectly matched layers (PMLs) were used on all the boundaries. However, anti-symmetric and symmetric boundary conditions were used for x-min and y-min for OS simulation. The effect of staircase approximation was addressed by reducing the mesh size around the features to 2 nm× 2 nm× 2 nm. The simulations were performed on a computer with 8-core calculation nodes, each carrying 4 GB memory. Gold dielectric constants were obtained from the literature [2].

III. Calculated electric field (E-field) distributions.

Figure S1 presents the calculated E-field distributions for the representative OS and SM architectures. Figure S1a depicts 4 Au nanospheres (100 nm diameter) touching each other with an in-between empty space of dimension 100 nm (along the E-field direction). Figure S1b shows the characteristic SM architecture, where a 0.8 μm diameter Au disk (corresponds to knobs) is sitting on an infinite Au fiber of diameter 1.5 μm.

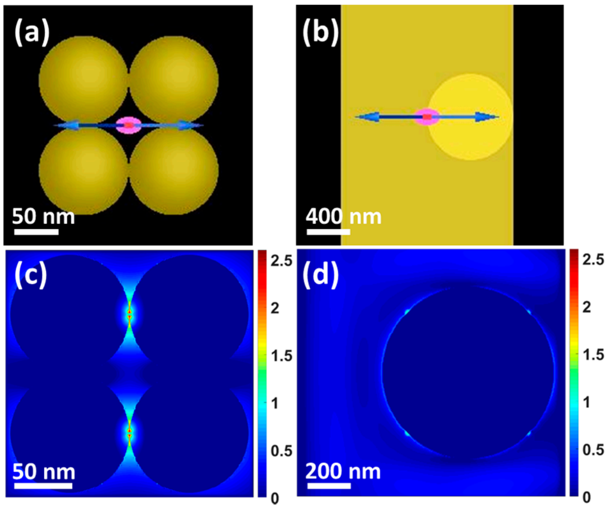

Figure S1. Representative (**a**) OS and (**b**) SM architectures. Calculated E-field distribution for (**c**) OS and (**d**) SM architectures. The incident wavelength is 785 nm.

As shown in Figure S1c and S1d, the E-field enhancement is larger for the aggregated nanospheres than that for the disk/fiber architecture. This qualitatively defends the better SERS performance of OS/IS region than the SM.

IV. Calculation of SERS enhancement factor (EF).

SERS EF is estimated according to the references 3 and 4 by comparing the 1575 cm^{-1} peak in the SERS spectrum to the 1585 cm^{-1} peak in normal Raman spectrum of the benzenethiol (BT) [3,4]. The EF can be written as

$$EF = \left(\frac{I_{SERS}}{I_{normal}}\right) \times \left(\frac{C_{normal}}{C_{SERS}}\right) \quad (6)$$

Here, I_{SERS} and I_{normal} represent the normalized (to power and integration time) count rates of the SERS and normal Raman signal, respectively. To obtain I_{normal}, BT is drop-casted on bare OS, IS, and SM regions without any Au coating. C_{SERS} and C_{normal} are the concentrations of the BT molecules in the SERS and normal Raman measurements. Table S1 enlists the calculated EF values for OS, IS, and SM regions.

Table S1. Calculation of SERS EF for egg OS, IS, and SM regions.

Region	C_{normal} (M)	Sample Power (mW)	Integration Time (s)	I_{normal} (photons s^{-1} mW^{-1})	EF
OS	4×10^{-2}	50	30	90	2.6×10^{6}
IS	4×10^{-2}	50	30	86	1.8×10^{6}
SM	4×10^{-1}	15	30	1040	1.5×10^{5}

References

1. Yee, K. Numerical solution of initial boundary value problems involving maxwell's equations in isotropic media. *IEEE Trans. Antennas* **1966**, *14*, 302–307.
2. Johnson, P.B.; Christy, R.-W. Optical constants of the noble metals. *Phys. Rev. B* **1972**, *6*, 4370.
3. Chen, J.; Su, H.; You, X.; Gao, J.; Lau, W.M.; Zhang, D. 3D TiO_2 submicrostructures decorated by silver nanoparticles as SERS substrate for organic pollutants detection and degradation. *Mater. Res. Bull.* **2014**, *49*, 560–565.
4. Lin, P.Y.; Hsieh, C.W.; Tsai, P.C.; Hsieh, S. Porosity-controlled eggshell membrane as 3D SERS-active substrate. *ChemPhysChem* **2014**, *15*, 1577–1580.
